# The effect of swimming exercise on adenine-induced kidney disease in rats, and the influence of curcumin or lisinopril thereon

**DOI:** 10.1371/journal.pone.0176316

**Published:** 2017-04-26

**Authors:** Badreldin H. Ali, Turan Karaca, Yousuf Al Suleimani, Mohammed Al Za'abi, Jamila Al Kalbani, Mohammed Ashique, Abderrahim Nemmar

**Affiliations:** 1 Department of Pharmacology and Clinical Pharmacy, College of Medicine and Health Sciences, Sultan Qaboos University, Al Khod, Oman; 2 Department of Histology-Embryology, Faculty of Medicine, University of Trakya, Balkan Campus, Edirne, Turkey; 3 Department of Physiology, College of Medicine and Health Sciences, United Arab Emirates University, Al Ain, United Arab Emirates; The University of Manchester, UNITED KINGDOM

## Abstract

Patients with chronic kidney disease (CKD) have been reported to benefit from different types of exercises. It has also been shown that the ACE inhibitor lisinopril, and the natural product curcumin are also beneficial in different models of CKD in rats. We assessed the influence of moderate swimming exercise (SE) on rats with adenine-induced CKD, and tested the possible effects of lisinopril and/or curcumin thereon using several physiological, biochemical, histopathological and immunohistochemical parameters. Rats (either sedentary or subjected to SE) were randomly divided into several groups, and given for five weeks either normal food or food mixed with adenine (0.25% w/w) to induce CKD. Some of these groups were also concomitantly treated orally with curcumin (75 mg/kg), or lisinopril (10 mg/kg) and were subjected to moderate SE (45 min/day three days each week). Rats fed adenine showed the typical biochemical, histopathological signs of CKD such as elevations in blood pressure, urinary albumin / creatinine ratio, and plasma urea, creatinine, indoxyl sulfate and phosphorus. SE, curcumin or lisinopril, given singly, significantly ameliorated all the adenine-induced actions. Administering curcumin or lisinopril with SE improved the histopathology of the kidneys, a salutary effect not seen with SE alone. Combining SE to the nephroprotective agents’ curcumin or lisinopril might offer additional nephroprotection.

## Introduction

Chronic kidney disease (CKD) is currently deemed to be a major and growing public health problem facing both developed [1] and developing [2] countries. It has been associated with poor health outcomes that include diminution in the quality and/or length of life [3]. CKD has a high prevalence of morbidity and mortality, principally due to cardiovascular dysfunction, neurohumoral impairment, and the development of end-stage renal disease (ESRD), which can cost annually more than US$ one trillion globally in clinical care [4, 5]. It is known that CKD can lead to reduced physical activity and an increased risk of cardiovascular disease (CVD) [6, 7]. It has been proven that a sedentary lifestyle increases the risk of CVD, which can be ameliorated by physical fitness [8, 9].

The polyphenolic compound curcumin) diferuloyl methane) is the main curcuminoid found in turmeric, and has been shown to significantly mitigate CKD in nephrectomized rats [10] and in adenine-induced CKD in rats [11]. This salutatory action was ascribed to several mechanisms (recently reviewed by Xia [12]).

It is known that hypertension is the most frequent cardiovascular complication in CKD as it predicts mortality, and is also a major determinant of progression of renal injury in humans and animals [13]. The angiotensin–converting enzyme inhibitor lisinopril is a commonly used antihypertensive and anti-proteinuric drug in humans, and is used as a reference drug when testing new compounds for the amelioration of diabetic nephropathy or CKD [14, 15].

Different aerobic exercises have been proven to improve renal and cardiac functions in individuals with CKD [7, 16], and in overweight rats with metabolic and cardiac dysfunction [17], and also in rats with experimental CKD [18, 19]. Appropriate exercise has been regarded as a possible tool for preventing, diminishing, or delaying CKD progression [20], and also for boosting patient's physical strength and quality of life [16]. Aerobic swimming exercise (SE) has been increasingly recommended as a non-pharmacological therapy for coronary heart disease, arterial hypertension, and obesity [21–24]. Therefore, obtaining data on the effects of SE (both alone, and in combination with medication) in animal models is of relevance for CKD patients [18, 19, 25, 26].

Because of the upsurge in recent decades of CKD incidence and its associated cardiovascular risks and damage [4], and in view of the lack of an effective cure for CKD so far [27], we thought it of importance to assess the effect of SE on a relevant rodent model of human CKD, where variable and modest effects was found [28]. We have also reported that administration of either an ACE inhibitor (lisinopril) or a natural product (curcumin), resulted in the amelioration of adenine-induced CKD in rats [11, 29]. Lisinopril is commonly used in the management of CKD in humans [27, 30].

Thus, in this study, we wished to ascertain whether the beneficial effects resulting from treatment of adenine-induced CKD in rats with either curcumin or lisinopril were enhanced by SE. It has been demonstrated that each of these treatments has a beneficial effect on its own, but whether SE enhanced the effect of either curcumin or lisinopril treatment needed to be determined.

## Materials and methods

### Animals

Female Sprague–Dawley rats (9–10 weeks old, weighing about 250 g) were kept in a room with a temperature of 22 ± 2°C, relative humidity of about 60%, with a 12 h light–dark cycle (lights on at 6:00). Animals were provided *ad libitum* with a standard pelleted chow diet containing 0.85% phosphorus, 1.12% calcium, 0.35% magnesium, 25.3% crude protein and 2.5 IU/g vitamin D3 (Oman Flour Mills, Muscat, Oman) and tap water.

### Ethics statement

Ethical approval for conducting the work was sought and obtained from Sultan Qaboos University (SQU) Animal Ethics Committee (SQU/AEC/2014–16). All procedures involving animals and their care were carried out in accordance with international laws and policies (EEC Council directives 2010/63/EU, 22 September, 2010 and NIH Guide for the Care and Use of Laboratory Animals, NIH Publications, 8th edition, 2011).

### Experimental design

Following an acclimatization period of seven days, rats (n = 78) were randomly distributed into 13 equal groups and treated for 35 consecutive days as follows:

The 1st group (Control) continued to receive the same diet without treatment.
The 2nd group was given adenine in the feed at a dose of 0.25%, w/w.
The 3rd group was treated as in group1, and orally treated with curcumin (75 mg/kg).
The 4th group was treated as in group1, but was subjected to SE.
The 5th group was treated as in group1, but was treated orally with lisinopril (10 mg/kg/day).
The 6th, 7th and 8th groups were fed adenine, and received curcumin, SE or lisinopril, respectively.
The 9th and 10th groups were subjected to SE and given either curcumin or lisinopril, respectively.
The 11th and 12th groups were treated with adenine and SE, and given either curcumin or lisinopril, respectively.
The 13th group was treated with adenine, curcumin, lisinopril and subjected to SE.

The treatments of the different groups are summarized in Table 1. The dose of curcumin and lisinopril were selected from previous experiments [11, 29].

**Table 1:** Study protocol and experimental design.

Groups/Treatment	1	2	3	4	5	6	7	8	9	10	11	12	13
	Control	A	C	SE	L	A+C	A+SE	A+L	C+SE	L+SE	A+C+SE	A+L+SE	A+C+L+SE
Drinking water (ad libitum)	√	√	√	√	√	√	√	√	√	√	√	√	√
Normal feed (ad libitum)	√	√	√	√	√				√	√			
2%^W/V^ CMC (2.0 mL/Kg/day)	√	√		√			√						
Swimming exercise (SE) on three alternate days/week (45 mins/day), 35 days				√			√		√	√	√	√	√
Adenine (A) 0.25%^W/V^, in feed, 35 days (ad libitum)		√				√	√	√			√	√	√
Oral curcumin (C, 75 mg/Kg/day), 35 days			√			√			√		√		√
Oral lisinopril (L, 10 mg/Kg/day), 35 days					√			√		√		√	√

### Swimming exercise training protocol

Rats were subjected first to a pre-SE for acclimation in an experimental swimming pool (25±1°C, water depth: 40 cm; radius 60 cm), as described before [18, 26]. Briefly, the pre-SE program included an acclimation period of four days (before the start of the experimental treatments), during which the rats were made to swim for 5 to 10 min, and then the training period was gradually increased to 20 min per day. Following the acclimation to swimming, the rats in the 4th, 7th, 9th, 10th, 11th, 12th, and 13th groups started to receive their respective treatments, and were also subjected to SE three days a week for 45 min each for the duration of the experiment (5 weeks).

### Treatments

One day before the last day of treatment, rats (which have been previously acclimatized on three occasions to housing singly in metabolic cages during the experimental period) were placed individually in metabolic cages to collect the urine voided in the last 24 h. Twenty-four hours after the end of the treatment, the rats were anesthetized with ketamine (75 mg/kg) and xylazine (5 mg/kg) intraperitoneally, and blood (about 5 mL) was collected and, together with obtained urine, centrifuged at 900 g at 4°C for 15 min to separate plasma. The plasma and urine were stored at −80°C pending analysis. The two kidneys were excised, blotted on filter paper and weighed. The right kidney and most of the left one were rapidly dipped in liquid nitrogen and kept frozen at −80°C for conducting biochemical tests and Western blotting. A small piece of the left kidney was placed in formol–saline for subsequent histopathological examination. The rats were then killed by an over dose of the anesthetics.

### Physiological and biochemical measurements

The body weights of all rats were recorded on a weekly basis during the experimental period, and the blood pressure (BP) was measured using a Blood Pressure Analysis System^™^ (BP-2000 SERIES *II*, Visitech Systems, Apex, NC, USA) as described before [29]. Plasma and urine osmolality were measured by the freezing point depression method (−70°C) using a Digimatic osmometer (Osmomat 3000, Gonotec GmbH, Berlin, Germany). Plasma neutrophil gelatinase-associated lipocalin (NGAL) activity was measured by an ELISA method using kits obtained from BioPorto Diagnostics (Gentofte, Denmark). Urinary *N*-acetyl-*β*-glucosaminidase (NAG) activity was measured by kits from Diazyme (Poway, CA, USA). Other plasma and urinary biochemical biomarkers of renal functions (creatinine, urea, creatinine clearance, calcium, and albumin) and plasma enzymes L-γ-glutamyltransferase (GGT), alkaline phosphatase (ALP), aspartate transaminase (AST) and alanine transaminase (ALT) were measured in an automated machine (Mindray BS-120 Chemistry Analyzer, Shenzhen, China). The concentrations of the uremic toxins phosphorus and indoxyl sulfate (IS) were measured in plasma by an automated method (Mindray BS-120 Chemistry Analyzer, Shenzhen, China), and by an HPLC method [31], respectively.

The plasma concentrations of tumor necrosis factor alpha (TNF-α), interleukin 6 (IL -6), interleukin 1β (IL -1β), adiponectin, and sclerostin were measured by ELISA methods, as detailed elsewhere [18, 32].

The supernatants of renal homogenates were separated and used for the measurement of total protein, catalase (CAT), glutathione reductase (GR), superoxide dismutase (SOD) and total antioxidant capacity (TAC), as described earlier [18, 32].

### Histopathology and immunohistochemistry

Light microscopic investigation of renal histology and scoring of damage were conducted as described before [18, 28, 33, 34]. Briefly, the kidney (4 μm sections) were cut and stained with hematoxylin (H & E), Masson Trichrome and Sirius Red stain. They were examined for necrosis and fibrosis by a specialist unaware of the treatments.

The sections were washed three times with PBS and incubated with biotinylated secondary antibody (Ultra Vision Detection System-HRP kit, Thermo, Fremont, USA), and then streptavidin peroxidase (Ultra Vision Detection System-HRP kit, Lab Vision, Fremont, USA) was given at room temperature for 20 min. 3-Amino-9-ethylcarbazole (AEC) was used as a chromogen, and the sections were counterstained with hematoxylin. The number of caspase 3–positive cells in each specimen was also scored. Cells with brown nuclear staining were considered positive, and the number of caspase 3–positive cells was counted in random high-power sections using a light microscope (Olympus BX51, Japan) and incorporating in a software analysis system (Argenit Kameram, ver. 2.11.5.1, Istanbul, Turkey). All the counts were converted to number of positive cells per unit area (mm^2^) [33, 34].

### Statistics

Values reported in this work were expressed as means ± SEM, and were analyzed with GraphPad Prism Version 5.03 for Windows software (Graphpad Software Inc., San Diego, USA). Comparisons between the different groups were conducted by analysis of variance (ANOVA), followed by Tukey’s multiple comparison tests. *P* values < 0.05 were considered significant.

## Results

### Physiological data

The general appearance of the rats with adenine-induced CKD was subjectively judged to be improved by treatment with curcumin, lisinopril, SE or combinations of these. The kidneys from control rats, or rats treated with either curcumin or lisinopril, with or without SE appeared normal. However, the kidneys of adenine treated rats were pale and with white crystals, similar to that described before [35].

The appearance of the kidneys of rats treated with adenine plus either curcumin, lisinopril, SE or combinations of these treatments was improved compared with the kidneys of rats treated with adenine alone. The physiological data of the groups in the experiment are shown in Table 2.

**Table 2:** Effect of swimming exercise (SE) along with curcumin (C) or lisinopril (L) treatments on some physiological parameters in rats with adenine (A)—induced chronic kidney disease.

Parameters/Treatment	Control	A	C	SE	L	A+C	A+SE	A+L	C+SE	L+SE	A+C+SE	A+L+SE	A+C+L+SE
Increase in body weight (%)	28.1±4.1	-8.5±1.1***	31.1±6.1	26.2±4.1	26.8±1.6	11.7±2.8*	-2.3±1.4***	3.9±0.6***	31.9±3.9	25.7±2.8	13.7±1.4*^†††^	4.5±0.5***	16.5±1.2^†††^
Relative kidney weight (%)	0.7±0	1.9±0.1***	0.8±0	0.7±0.2	0.7±0	1.1±0***	1.7±0.1***	1.6±0***	0.7±0	0.7±0	1.0±0.1**^††^	1.6±0.1***^††^	0.9±0.1^†††^
Water intake (mL)	20.4±1.6	52.1±6.4***	25.8±1.8	18.0±2.0	22.0±1.8	46.6±2.0***	47.1±2.9***	35.6±3.1**	22.5±1.8	19.6±1.6	40.4±2.5***	44.6±2.5***	37.9±3.8**^†^
Urine output (mL)	7.8±1.1	38.2±1.9***	6.8±0.9	6.2±0.4	9.2±0.7	27.8±1.9***	34.0±2.5***	30.1±0.7***	9.7±1.4	8.8±1.1	26.8±3.1***^††^	32.0±2.7***	25.2±1.5***^†††^

Values in the table are means ± SEM (n = 6)

Chronic kidney disease was induced by inclusion of A in the feed (0.25%^W/V^, for 35 days). C (75mg/Kg) or L (10mg/Kg) were given to rats concurrently by oral gavage. SE was performed for selected groups for 45 mins/day, three days/week. On day 35 the rats were placed in metabolic cage to collect urine.

Significance of different groups vs control group, where:

*P < 0.05,

**P < 0.001,

***P < 0.0001.

Significance of the group treated with A alone vs its corresponding groups treated with A and subjected to SE, where:

^†^P < 0.05,

^††^P < 0.001,

^†††^P < 0.0001.

Adenine treatment significantly decreased the growth of rats, and increased the relative kidney weight, as well as the water intake and urine output. SE, treatment with either curcumin or lisinopril, and their various combinations increased the body weights of rats to a similar level to that of the controls, and did not significantly affect either their water intake or urine output. However, when curcumin was given concomitantly with adenine, the actions of adenine on these parameters was significantly mitigated (*F* value 21.29 to 75.83, *P* < 0.05 to *P* < 0.0001). When either SE or lisinopril was given concomitantly with adenine, the adenine-induced drop in growth appeared to be ameliorated, but not statistically significantly. Neither curcumin nor SE affected either water intake or urinary output of adenine treated rats.

**Fig 1:**
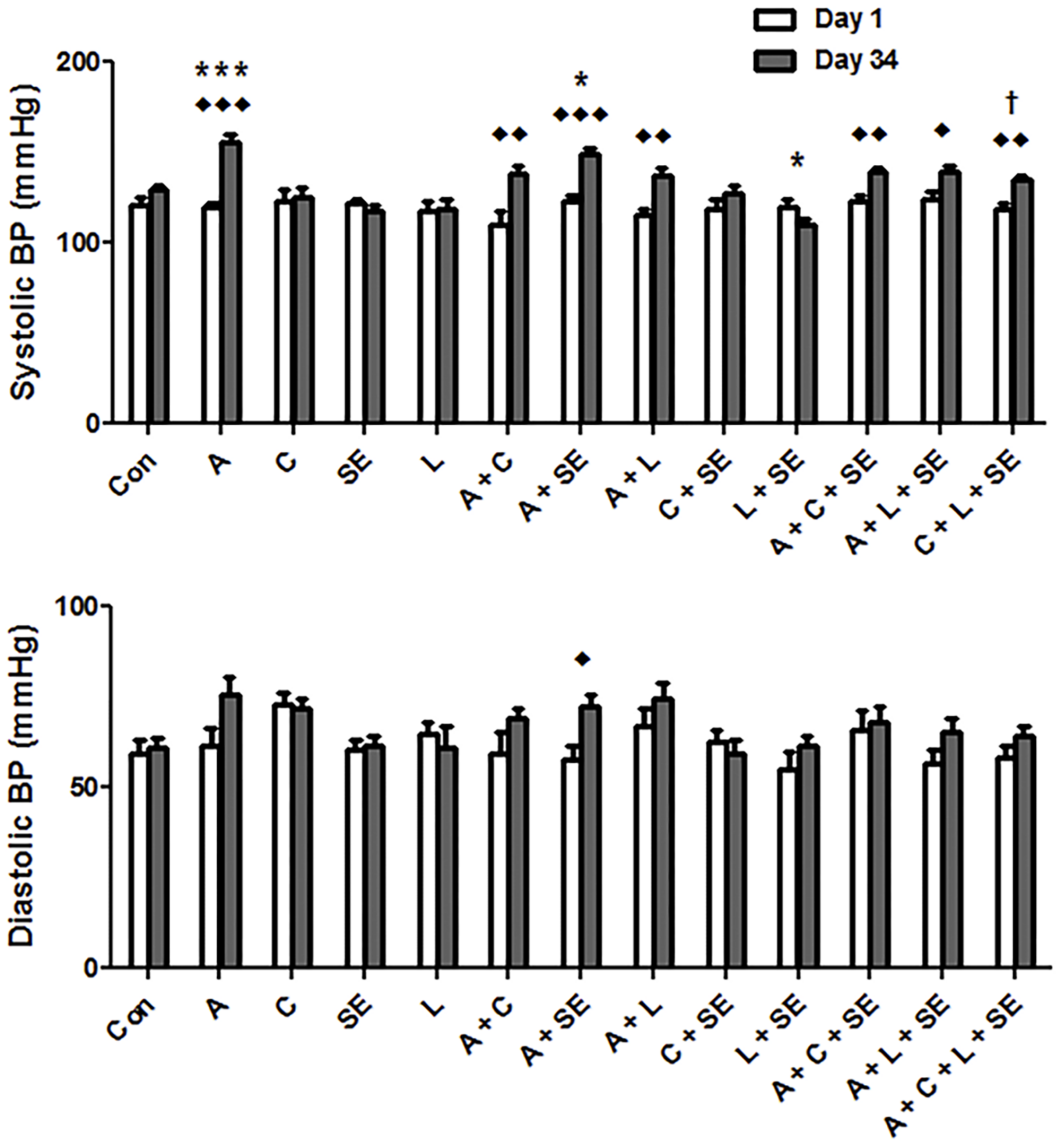
Systolic (SBP) and diastolic blood (DBP) pressure data. Control (Con) rats, rats treated with adenine (A) in the feed (0.25%^W/V^), curcumin (C, 75 mg/kg), or lisinopril (L, 10 mg/kg), separately or in combination, and subjected to swimming exercise (SE). The measurements were conducted on the first day of the experiment and one day before sacrificing the animals. Each column and vertical bar represents mean ± SEM (n = 6). ◆ Denotes significance of Day 1 vs. Day 34 of each experimental group: where ◆*P* < 0.05, ◆◆*P* < 0.001, ◆◆◆*P* < 0.0001. * denotes significance of different groups vs. Control group: where *P < 0.05, ***P < 0.0001. ^†^ Denotes significance of the group treated with A alone vs. its corresponding groups treated with A and subjected to SE: where ^†^*P* < 0.05.

Fig 1 shows the effect of adenine, curcumin, lisinopril, SE and their combinations on the systolic and diastolic BP taken on day 1 and day 34 of the treatment. Adenine treatment induced a significant (*F* value 10.53, *P* < 0.0001) elevation of the systolic BP, and an insignificant rise in diastolic BP. The adenine-induced rise in systolic BP was mitigated curcumin, lisinopril, SE or their combinations were given, but these values were still significantly higher than in control rats, or rats treated with curcumin, lisinopril, SE or their combinations in the absence adenine.

Diastolic BP was not significantly affected in any of the groups except in the group treated with adenine and subjected to SE, where there was a significant increase (*F* value 4.03, *P* < 0.05).

### Plasma and urinary biochemical parameters

As shown in Table 3, adenine treatment significantly increased plasma concentrations of urea, creatinine, IS and phosphorus, and significantly decreased creatinine clearance and calcium concentrations (*F* value 5.52 to 41.22, *P* > 0.0001). Treatments with curcumin, lisinopril or SE, on their own, or in combination, did not significantly affected the concentrations of these plasma constituents. SE alone caused a slight and statistically insignificant effect on the above values in adenine-treated rats. Co-administration of adenine with either curcumin, lisinopril, or their combinations significantly improved the plasma renal function tests measured.

**Table 3:** Effect of swimming exercise (SE) along with curcumin (C) or lisinopril (L) treatments on renal functions tests in plasma of rats with adenine (A) -induced chronic kidney disease.

Test/Treatment	Control	A	C	SE	L	A+C	A+SE	A+L	C+SE	L+SE	A+C+SE	A+L+SE	A+C+L+SE
Urea (mmol/L)	5.7±0.2	24.1±1.7***	5.1±0.2	6.4±0.3	5.6±0.4	17.1±1.2***	21.9±2.1***	19.7±2.0***	5.9±0.5	6.1±0.6	18.2±1.3***	20.7±1.6***	17.1±1.2***^††^
Creatinine (μmol/L)	18.5±1.1	75.1±5.3***	19.0±0.5	19.7±1.7	20.4±1.0	54.9±5.6***	70.9±7.2***	87.0±6.0***	2.3±3.0	21.8±3.4	49.2±3.0***^††^	65.1±7.2***	43.3±3.5**^†††^
Creatinine clearance (mL/min)	2.7±0.3	0.3±0***	3.4±0.2	2.1±0.2	3.7±0.4*	0.7±0.1***	0.6±0.1***	0.5±0.1***	3.1±0.2	3.3±0.4	1.8±0.2^†††^	1.1±0.1***	3.1±0.2^†††^
Indoxyl sulfate (% control)	100.0±0	477.5±65.9***	100.2±14.8	109.3±9.9	154.1±8.3	Not done	247.4±30.5**^†††^	Not done	114.6±4.3	157.4±16.6	132.3±8.4^†††^	164.4±16.6^†††^	177.8±12.1^†††^
NGAL (ng/mL)	34.1±2.1	138.0±7.8***	23.0±5.1	29.0±3.8	78.3±8.9**	87.7±8.6***	114.1±10.5***	132.3±8.8***	28.2±3.0	59.3±5.8	62.2±4.9^†††^	108.8±13.0***	67.4±6.0^†††^
Phosphorous (mmol/L)	2.2±0.1	4.0±0.2***	2.1±0.2	2.9±0.3	2.4±0.1	2.9±0.3	3.6±0.3**	3.0±0.3	2.3±0.1	2.7±0.3	2.6±0.2^††^	3.0±0.3	2.5±0.2^††^
Calcium (mmol/L)	2.7±0.1	0.7±0.1***	2.7±0.2	2.6±0.3	2.4±0.2	1.1±0.1***	0.9±0.1***	1.2±0.1***	2.9±0.2	2.3±0.2	1.5±0.1***	1.4±0.2***	1.7±0.2*^††^

Values in the table are means ± SEM (n = 6)

Chronic kidney disease was induced by inclusion of A in the feed (0.25%^W/V^, for 35 days). C (75mg/Kg) or L (10mg/Kg) were given to rats concurrently by oral gavage. SE was performed for selected groups for 45 mins/day, three days/week. On day 35 the rats were placed in metabolic cage to collect urine.

NGAL: neutrophil gelatinase-associated lipocalin

Significance of different groups vs control group, where:

*P < 0.05,

**P < 0.001,

***P < 0.0001.

Significance of the group treated with A alone vs its corresponding groups treated with A and subjected to SE, where:

^†^P < 0.05,

^††^P < 0.001,

^†††^P < 0.0001.

The activities of the plasma enzymes ALT, ALP, GGT and AST were all significantly raised by adenine feeding (Table 4). Treatments with curcumin, lisinopril or SE, on their own, or in combination, did not significantly affect the activities of these enzymes. Addition of curcumin, lisinopril or SE to adenine treatment, significantly ameliorated the action of adenine effect on the enzyme activities. However, they did not significantly return them to the control values.

**Table 4:** Effect of swimming exercise (SE) along with curcumin (C) or lisinopril (L) treatments on the concentration of some plasma enzymes and urinary constitutes in rats with adenine (A) -induced chronic kidney disease.

Constitute/Treatment	Control	A	C	SE	L	A+C	A+SE	A+L	C+SE	L+SE	A+C+SE	A+L+SE	A+C+L+SE
Alanine aminotransferase (IU/L)	38.8±2.5	92.2±5.8***	37.7±3.6	35.1±4.3	44.5±2.0	62.3±2.1*	77.7±5.5***	65.1±2.0**	42.8±5.0	47.2±6.5	59.7±5.9^†††^	74.9±6.4***	55.9±4.5^†††^
Alkaline phosphatase (IU/L)	85.0±5.8	382.5±21.2***	85.0±6.9	75.8±7.1	90.7±5.2	142.3±14.1	231.0±25.8***^†††^	174.9±12.9***	79.6±9.4	96.0±10.1	110.2±8.4^†††^	170.3±19.6**^†††^	98.6±7.5^†††^
γ-glutamyl transferase (IU/L)	1.0±0.1	5.8±0.3***	1.3±0.2	1.3±0.2	1.2±0.2	3.0±0.3***	4.8±0.3***	2.5±0.2***	1.5±0.2	1.0±0.1	2.3±0.2**^†††^	2.7±0.3***^†††^	1.9±0.1^†††^
Aspartate aminotransferase (IU/L)	62.1±4.3	208.4±13.9***	69.2±5.1	72.6±6.5	71.2±4.9	162.1±8.1***	188.2±17.4***	171.0±16.0***	63.3±3.7	71.9±9.4	153.7±13.9***	149.3±16.4***	117.3±9.4*^†††^
Urinary N-acetyl-β-D-glucosaminidase (IU/L)	4.3±0.3	29.2±1.6***	6.6±0.7	6.5±0.9	12.1±1.8	23.3±1.0***	25.0±1.9***	24.0±0.4***	5.5±0.8	8.4±1.4	16.1±1.4**^††^	21.0±1.6***	18.0±1.9***^†^
Urinary albumin/creatinine ratio	0.1±0	4.6±0.2***	0.1±0	0.1±0	0.1±0	Not done	1.5±0.2***^†††^	Not done	0.1±0	0.1±0	0.5±0***	0.8±0.1***^†††^	0.2±0^†††^
Urine osmolality (mOsmol/Kg)	1508.3±46.4	261.5±12.9***	1864.7±51.5***	1315.8±77.1	1747.3±54.2	693.3±42.8***	366.5±27.4***	493.2±28.5***	1665.2±35.4	1476.5±103.2	714.3±26.3***^†††^	641.5±50.8***^†††^	949.8±65.3***^†††^

Values in the table are means ± SEM (n = 6)

Chronic kidney disease was induced by inclusion of A in the feed (0.25%^W/V^, for 35 days). C (75mg/Kg) or L (10mg/Kg) were given to rats concurrently by oral gavage. SE was performed for selected groups for 45 mins/day, three days/week. On day 35 the rats were placed in metabolic cage to collect urine.

Significance of different groups vs control group, where:

*P < 0.05,

**P < 0.001,

***P < 0.0001.

Significance of the group treated with A alone vs its corresponding groups treated with A and subjected to SE, where:

^†^P < 0.05,

^††^P < 0.001,

^†††^P < 0.0001.

Table 4 also summarizes the effect of adenine, curcumin, lisinopril, SE and their combinations on some urinary biomarkers. Adenine significantly increased the activity of NAG, and the creatinine/albumin ratio, and decreased osmolality. Curcumin, lisinopril and SE, significantly abated this action. Lisinopril was less effective than SE or curcumin in abating the urinary creatinine/albumin ratio.

However, the three were more or less equal in their effect on NAG and osmolality.

Combinations of the curcumin, lisinopril, SE did not afford any benefit to the action when each treatment applied alone.

Table 5 summarizes the effect of adenine, curcumin, lisinopril, SE and their combinations on some antioxidant indices in kidney of the rats with adenine-induced CKD. Adenine feeding significantly reduced the activities or concentrations of the measured indices. Curcumin, lisinopril, and SE, given separately, significantly ameliorated these actions (and in some cases to above the control values). When each of these was given together with adenine, the reductions induced by adenine were mitigated, and in this regard, curcumin was the most effective and SE was the least effective. When adenine was given together with curcumin or with SE, no significant additional benefit was noted, except for SOD activity.

**Table 5:** Effect of swimming exercise (SE) along with curcumin (C) or lisinopril (L) treatments on some antioxidant indices in rats with adenine (A) -induced chronic kidney disease.

Parameter/Treatment	Control	A	C	SE	L	A+C	A+SE	A+L	C+SE	L+SE	A+C+SE	A+L+SE	A+C+L+SE
Total antioxidant capacity (mM)	0.5±0	0.2±0***	0.4±0	0.5±0	0.4±0.1	0.2±0***	0.2±0***	0.2±0***	0.4±0	0.4±0.1	0.3±0**	0.2±0***	0.4±0^††^
Superoxide dismutase (% control)	100.0±0	36.7±2.1***	92.1±3.7	108.0±7.1	106.6±5.2	64.6±2.4***	47.9±3.4***	55.0±1.8***	99.4±8.7	102.4±6.9	72.0±3.6^†††^	57.0±6.3**^†††^	81.6±4.4^†††^
Catalase (nm/min/mL)	0.3±0	0.2±0***	0.4±0	0.4±0	0.3±0	0.2±0	0.2±0**	0.2±0***	0.4±0.1	0.3±0	0.2±0	0.2±0*	0.3±0^†^
Total glutathione (μM)	5.2±0.3	2.4±0.2**	4.1±0.5	4.4±0.5	7.0±0.7	4.6±0.3	3.0±0.3	6.4±0.6	5.1±0.5	6.2±0.6	5.2±0.6^††^	6.1±0.5**^†††^	6.3±0.5^†††^

Values in the table are means ± SEM (n = 6)

Chronic kidney disease was induced by inclusion of A in the feed (0.25%^W/V^, for 35 days). C (75mg/Kg) or L (10mg/Kg) were given to rats concurrently by oral gavage. SE was performed for selected groups for 45 mins/day, three days/week. On day 35 the rats were placed in metabolic cage to collect urine.

Significance of different groups vs control group, where:

*P < 0.05,

**P < 0.001,

***P < 0.0001.

Significance of the group treated with A alone vs its corresponding groups treated with A and subjected to SE, where:

^†^P < 0.05,

^††^P < 0.001,

^†††^P < 0.0001.

Table 6 depicts the effects of curcumin, lisinopril, SE and their combinations on some cytokines in the plasma of rats with adenine-induced CKD. Adenine treatment significantly increased the plasma concentrations of adiponectin, cystatin, TNF-α and IL-6, and significantly decreased that of sclerostin. Curcumin and SE significantly and markedly abated these actions, and reversed some of these indices to above the control values. Concomitant administration of curcumin and SE lessened the salutary action of each given singly.

**Table 6:** Effect of swimming exercise (SE) along with curcumin (C) or lisinopril (L) treatments on some cytokines in the plasma of rats with adenine (A) -induced chronic kidney disease.

Parameter/Treatment	Control	A	C	SE	L	A+C	A+SE	A+L	C+SE	L+SE	A+C+SE	A+L+SE	A+C+L+SE
Adiponectin (μg/mL)	3.6±0.2	7.7±0.4***	2.8±0.4	4.2±0.6	5.4±0.4	5.2±0.4	6.9±0.4***	5.9±0.5*	3.7±0.4	4.8±0.6	4.7±0.2^†††^	5.7±0.4	4.9±0.4^†††^
Sclerostin (ng/mL)	0.7±0	0.2±0***	0.5±0***	0.5±0	0.3±0***	0.4±0***	0.3±0***^†^	0.2±0***	0.4±0***	0.5±0.1*	0.5±0**^†††^	0.3±0***^†^	0.4±0.1***^†††^
Cystatin C (μg/mL)	0.9±0	2.8±0.1***	0.9±0.1	0.8±0.1	0.7±0	1.9±0.1***	1.9±0.1***^†††^	1.6±0.1***	0.8±0.1	0.9±0.1	1.5±0.1**	1.7±0.2***^†††^	1.3±0.1^†††^
TNF-α (pg/mL)	17.3±0.8	75.4±4.0***	12.5±1.7	21.9±1.5	17.5±0.5	51.3±3.3***	64.2±5.6***	56.4±3.5***	17.1±2.1	28.8±2.7	42.1±5.1***^†††^	50.9±2.7***^†††^	38.9±3.0***^†††^
IL-6 (pg/mL)	36.7±3.0	162.2±9.2***	37.8±3.8	22.2±2.7	47.2±7.1	138.9±7.2***	126.7±10.3***	140.6±14.9***	44.4±6.4	35.0±3.8	97.8±9.4***^†††^	128.3±14.4***	105.6±8.7***^††^

Values in the table are means ± SEM (n = 6)

Chronic kidney disease was induced by inclusion of A in the feed (0.25%^W/V^, for 35 days). C (75mg/Kg) or L (10mg/Kg) were given to rats concurrently by oral gavage. SE was performed for selected groups for 45 mins/day, three days/week. On day 35 the rats were placed in metabolic cage to collect urine.

Significance of different groups vs control group, where:

*P < 0.05,

**P < 0.001,

***P < 0.0001.

Significance of the group treated with A alone vs its corresponding groups treated with A and subjected to SE, where:

^†^P < 0.05,

^††^P < 0.001,

^†††^P < 0.0001.

### Histopathological findings

**Fig 2:**
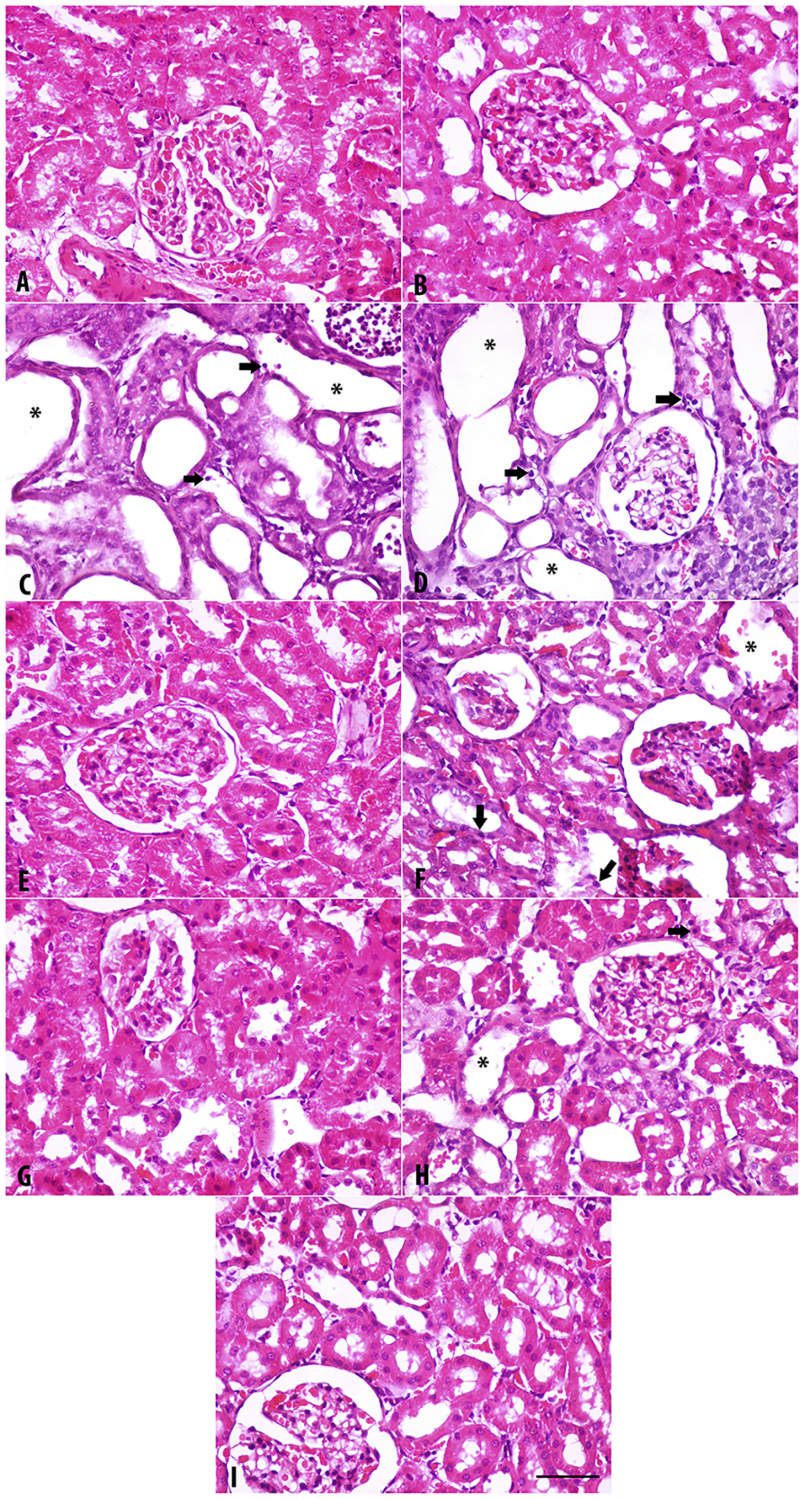
Representative light microscopy images of H&E stained kidney sections. Sections were taken from rats treated for 35 days with adenine (0.25%^W/V^ in feed), swimming exercise, curcumin (75 mg/kg), lisinopril (10 mg/kg) or a combination of these treatments. A. Control; B. Swimming group; C. Adenine; D. Adenine + Swimming; E. Curcumin + Swimming; F. Curcumin + Adenine + Swimming; G. Lisinopril + Swimming; H. Lisinopril + Adenine + Swimming; I. Curcumin + Lisinopril + Adenine + Swimming. Arrows: Necrotic nucleus; *: Dilated tubule. Magnification: 400X. Bar: 50 μm.

**Fig 3:**
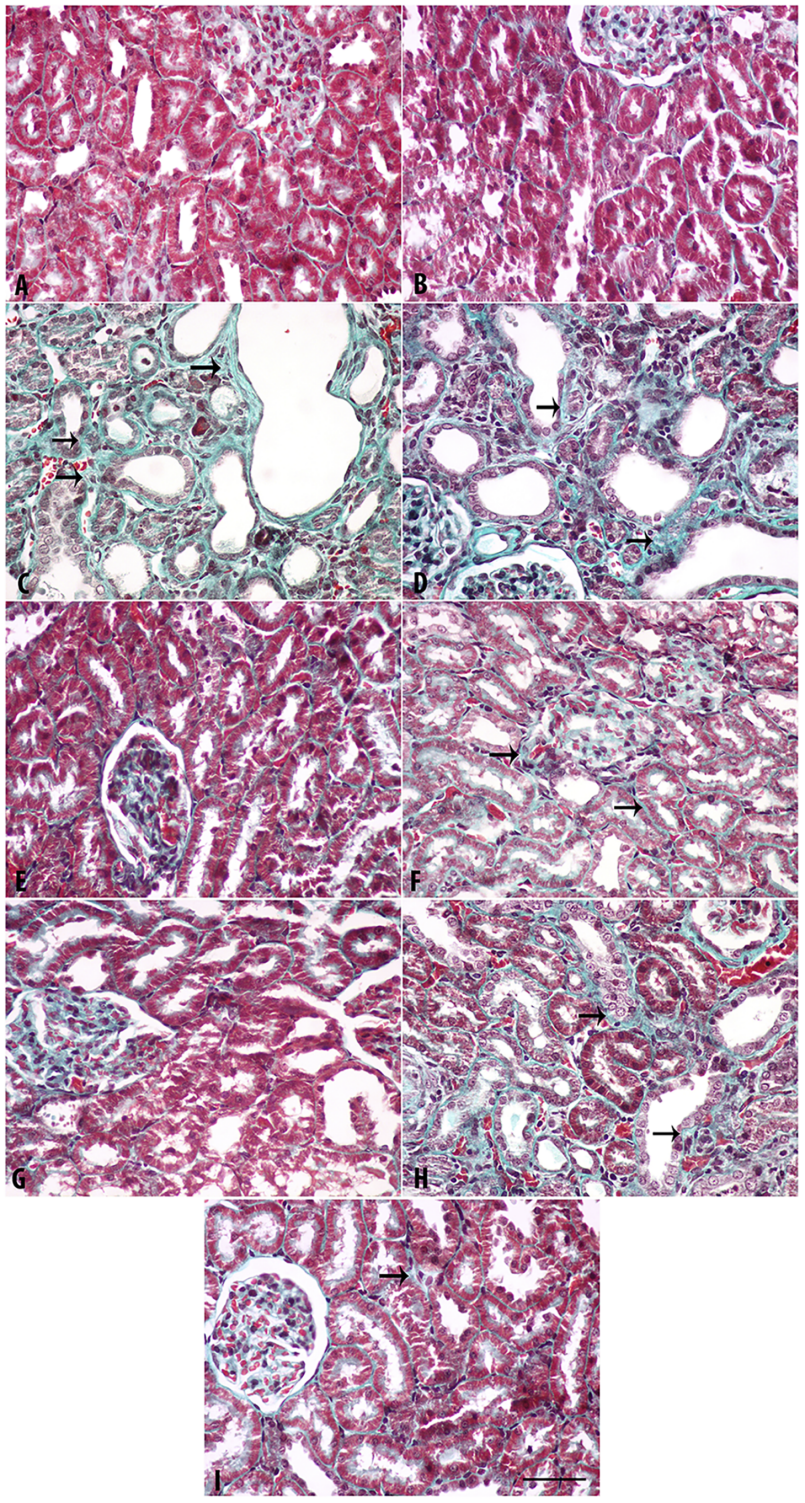
Representative light microscopy images of Masson Trichrome (MT) stained kidney sections. Sections were taken from rats treated for 35 days with adenine (0.25%^W/V^ in feed), swimming exercise, curcumin (75 mg/kg), lisinopril (10 mg/kg) or a combination of these treatments. A. Control; B. Swimming group; C. Adenine; D. Adenine + Swimming; E. Curcumin + Swimming; F. Curcumin + Adenine + Swimming; G. Lisinopril + Swimming; H. Lisinopril + Adenine + Swimming; I. Curcumin + Lisinopril + Adenine + Swimming. Arrows: Fibrosis. Magnification: 400X. Bar: 50 μm.

**Fig 4:**
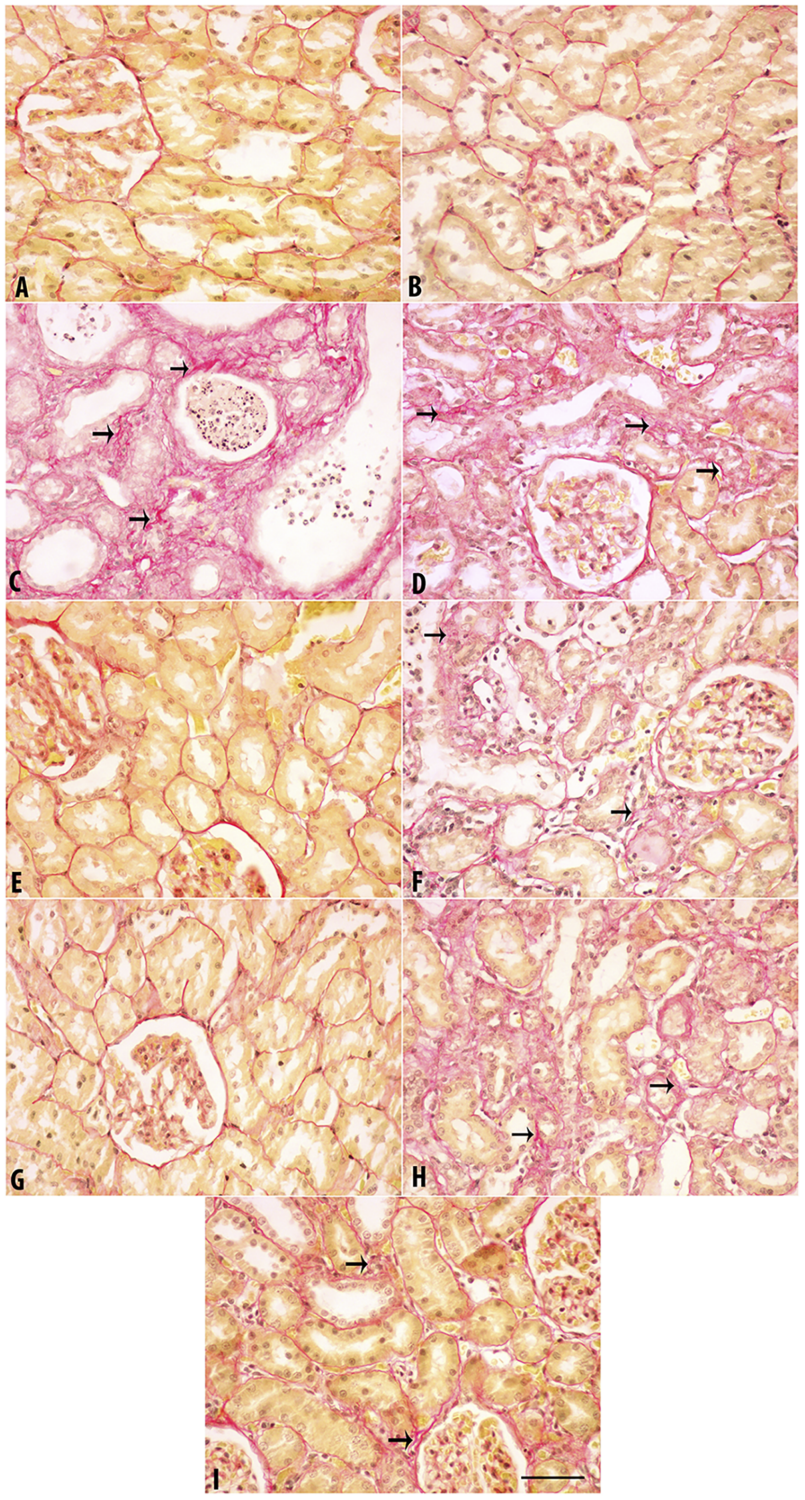
Representative light microscopy images of Sirius Red (SR) stained kidney sections. Sections were taken from rats treated for 35 days with adenine (0.25%^W/V^ in feed), swimming exercise, curcumin (75 mg/kg), lisinopril (10 mg/kg) or a combination of these treatments. A. Control; B. Swimming group; C. Adenine; D. Adenine + Swimming; E. Curcumin + Swimming; F. Curcumin + Adenine + Swimming; G. Lisinopril + Swimming; H. Lisinopril + Adenine + Swimming; I. Curcumin + Lisinopril + Adenine + Swimming. Arrows: Fibrosis. Magnification: 400X. Bar: 50 μm.

The results are depicted in Fig 2 (H & E staining), Fig 3 (Masson Trichrome staining) and Fig 4 (Sirius Red staining). The last two stains were used to evaluate fibrosis in renal tissue.

With the three stains, the control rats and rats in the groups subjected to SE, or were given curcumin + SE, as well as lisinopril + SE showed normal kidney architecture and histology. In rats fed adenine and the adenine + SE group, intensive tubular necrosis, tubular dilatation, tubular cast formation, necrotic nuclei and mononuclear leucocytes infiltration were seen. In animals that were given curcumin + adenine and were subjected to SE (Group F), or lisinopril + adenine + SE (Group H), or curcumin + lisinopril + adenine + SE (Group I), less histopathological changes were seen. In particular, tubular dilation, cast formation and mononuclear infiltration were lower in group I.

Table 7 summarizes the histopathological findings in the groups examined. The degrees of tubular necrosis, interstitial fibrosis, tubular cell “hyaline droplet formation”, hyaline cast formation and mononuclear cell infiltration were highest in the adenine-fed group. The groups treated with adenine + curcumin and subjected to SE showed a similar degree of damage to that treated with adenine + lisinopril and subjected to SE. Both were significantly less than those seen in rats treated with adenine alone.

**Table 7:** Effect of swimming exercise (SE) along with curcumin (C) or lisinopril (L) treatments on some histopathological parameters in rats with adenine (A) -induced chronic kidney disease.

Groups/Treatment	Control	A	C	SE	L	A+SE	C+SE	L+SE	A+C+SE	A+L+SE	A+C+L+SE
Tubular necrosis	0.2±0	3.2±0.3***	0±0	0±0	0±0	2.8±0.2***	0.2±0	0.2±0	1.7±0.2***^†††^	1.8±0.3***^†††^	1.3±0.2***^†††^
Interstitial fibrosis	0.2±0	2.8±0.5***	0±0	0.2±0	0±0	2.7±0.2***	0.2±0	0±0	1.5±0.2**^††^	1.7±0.2***^††^	1.2±0.3*^†††^
Tubular cell “hyaline droplet formation”	0±0	2.3±0.3***	0±0	0.2±0	0±0	2.2±0.2***	0±0	0.2±0	1.3±0.3***^†^	1.2±0.2**^††^	0.8±0.3^†††^
Hyaline cast formation	0±0	1.7±0.3***	0±0	0±0	0±0	1.7±0.2***	0.0±0	0.2±0	1.5±0.3***	1.5±0.2***	0.7±0.2^†^
Mononuclear cell infiltration	0±0	2.7±0.2***	0±0	0.2±0	0±0	2.2±0.3***	0.2±0	0±0	1.7±0.2***^††^	1.5±0.2***^†††^	0.8±0.3*^†††^

Values in the table are means ± SEM (n = 6)

Chronic kidney disease was induced by inclusion of A in the feed (0.25%^W/V^, for 35 days). C (75mg/Kg) or L (10mg/Kg) were given to rats concurrently by oral gavage. SE was performed for selected groups for 45 mins/day, three days/week. On day 35 the rats were placed in metabolic cage to collect urine.

Significance of different groups vs control group, where:

*P < 0.05,

**P < 0.001,

***P < 0.0001.

Significance of the group treated with A alone vs its corresponding groups treated with A and subjected to SE, where:

^†^P < 0.05,

^††^P < 0.001,

^†††^P < 0.0001.

### Immunohistochemical findings

**Fig 5:**
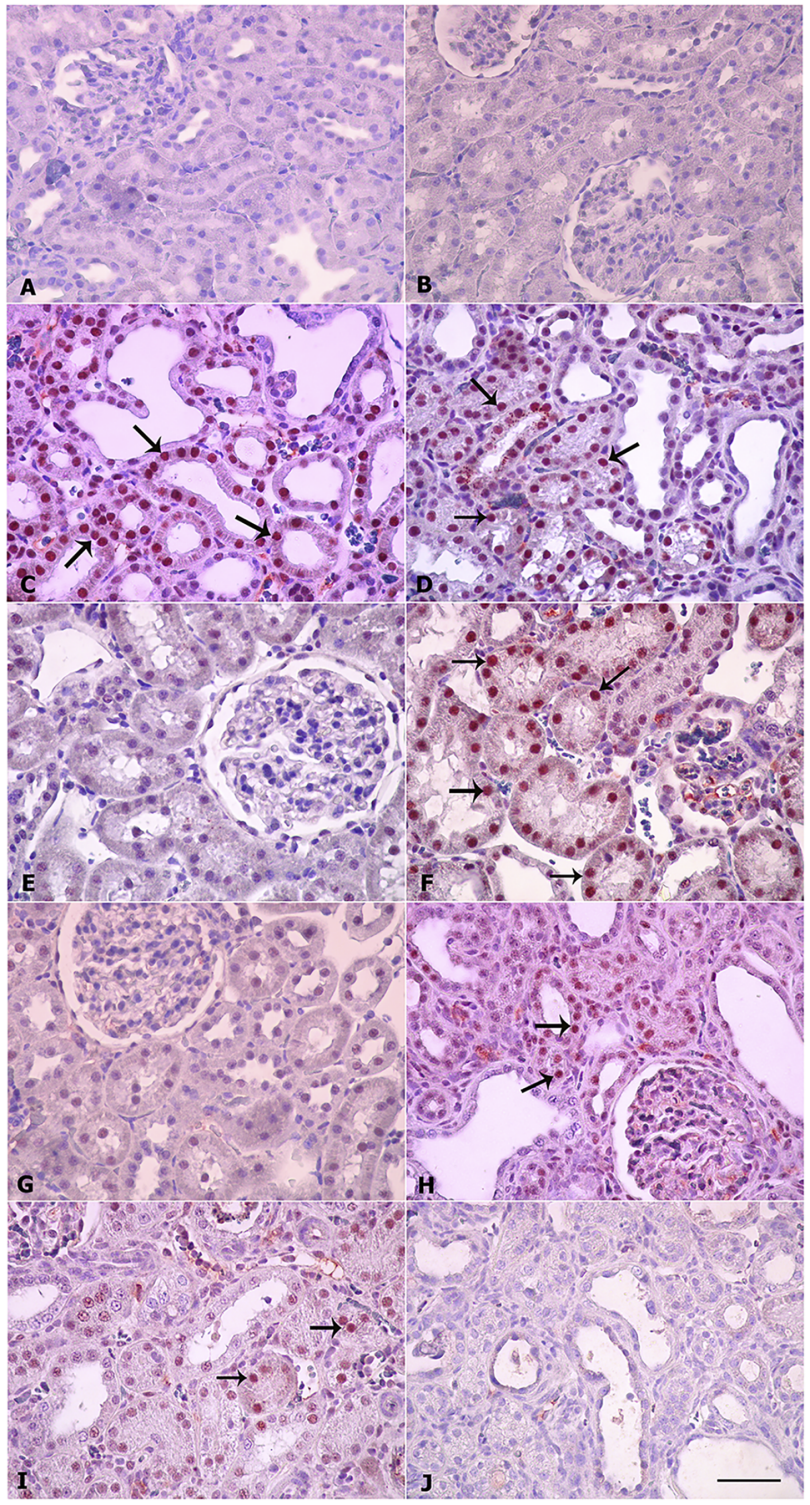
Immunohistochemistry of caspase-3 in kidney sections. Sections were made from kidneys of rats treated for 35 days with adenine (0.25%, w/w in feed), swimming exercise, curcumin (75 mg/kg), lisinopril (10 mg/kg) or a combination of these treatments. A. Control; B. Swimming group; C. Adenine; D. Adenine + Swimming; E. Curcumin + Swimming; F. Curcumin + Adenine + Swimming; G. Lisinopril + Swimming; H. Lisinopril + Adenine + Swimming; I. Curcumin + Lisinopril + Adenine + Swimming. Bar: 50 μm.

Caspase 3–positive cell count was made in 100 different areas from each group and is depicted in Fig 5. In the groups A, B, E and G no caspase 3–positive cells were observed. The numbers of caspase 3–positive cells were highest in the adenine-treated rats (group C) and adenine plus swimming group (group D). Curcumin and lisinopril treatments decreased the number of caspase 3–positive cells. The lowest number was seen in the groups given a combination of curcumin, lisinopril and subjected to SE.

## Discussion

To our knowledge this is probably the first study to investigate the possible interaction between SE and treatment with curcumin in rats with adenine-induced CKD. We showed here that although few of the studied parameters were enhanced by combining SE with curcumin and/or lisinopril, but the histopathological improvement seen with curcumin might suggest a salutary additional benefit of such combination in this animal model of CKD. As previously reported [35, 36], adenine-fed rats had lower final body weights, probably due to uremia, polyuria and dehydration, but also the poor palatability of adenine.

Curcumin, given concomitantly with adenine, was effective in significantly ameliorating the decrease in body weight, as well as the other actions. Adenine feeding also significantly increased systolic e BP, as was reported previously [29]. Neither curcumin nor lisinopril or SE was able to significantly abate this rise in the systolic BP.

Interestingly, however, among all the treatment groups, rats that have been treated with lisinopril and concomitantly subjected to SE had significantly lower systolic BP at the end of the experiment. The salutary action of aerobic exercise when combined with lisinopril on systolic BP in rats has been reported before [37]. The rise in BP in adenine-induced CKD can be explained by, among other related factors, the oxidative stress that occurs in this model. For example, it has been shown that oxidative stress upregulates renal angiotensin II type 1 receptor leading to hypertension [38].

Curcumin has been shown by several workers to mitigate renal dysfunction in a number of *in vivo* and *in vitro* models of renal diseases and conditions that include gentamicin and cisplatin-induced acute kidney injury [39, 40], CKD induced by subtotal nephrectomy [10] and by adenine feeding [11]. Recently, Bugyei-Twum et al. [41] reported that a novel formulation of curcumin (theracurmin) was successful in preventing cardiac fibrosis and diastolic dysfunction induced in rats with CKD by subtotal nephrectomy. In this work, curcumin was effective in significantly mitigating the actions of adenine on the measured plasma and urinary renal biomarkers and histology. In this regard, its ameliorative actions were broadly similar to those of the standard drug lisinopril, and slightly better than SE on its own. Lisinopril (and other angiotensin converting enzyme inhibitors such as enalapril) has been used in human patients [27] and rats [29] with CKD. Angiotensin II is known to increase in CKD and induce oxidative stress [42], and the ACE inhibitor lisinopril is expected to reverse this action.

Oxidative stress and inflammation are known to be constant features in CKD, and may be the basis of the ensuing hypertension and other consequences [43, 44]. Adenine feeding induced the expected and previously reported oxidative stress and inflammatory actions on the markers in plasma and kidney homogenates. Curcumin, being a strong antioxidant and anti-inflammatory agent, induced significant amelioration in all these measurements, which were in some cases to a level above that of the control. However, combining SE with either curcumin or lisinopril treatments did not offer any additional benefit in abating the actions of adenine. As a matter of fact, in most cases, the combination lessened the salutary effect of either SE or curcumin given singly. The reason for this latter action is not certain.

The mechanism by which SE can affect CKD is not known with certainty, but it has been hypothesized that the basis of the purported benefits is probably multifactorial [16] and include beneficial effect of SE on the oxidative status of the tissues. Different exercise modalities, including SE, have been reported to be beneficial in CKD and its cardiovascular and other related complications in human patients [45–48] and experimental animals [18, 26]. In addition to correcting the imbalance between the generation of reactive oxygen species and the antioxidant defense systems, the basis of these purported benefits may also be related to delaying the occurrence of hypertension and/or decreasing its level by central and peripheral neurohumoral mechanisms [49]. However, in a recent systematic review, it has been reported that the strongest evidence obtained was for the salutatory actions of aerobic exercise on improving physical fitness, muscular strength and quality of life in patients on dialysis [6]. Till now, a confirmed evidence on established benefits of exercise in patients at earlier stages of CKD and following renal transplantation is still not available. This may explain the lack of strong evidence for a salutatory action of SE on most of the measured indices of our model of CKD.

Although the reports in the literature regarding the impact of exercise on inflammation and oxidative stress are at variance, moderate SE is believed to be effective in preventing inflammation and oxidative damage in tissues of rats [18], but in both humans and rats severe/acute exercise has been shown to produce the reverse [50]. In our recent experiments employing moderate SE in rats, we observed that it did not significantly alter the renal concentration/activity of the measured indices of oxidative stress (except SOD activity, which was increased), probably reflecting the efficacy of the defensive antioxidant oxidative abilities in these animals. As reported before, adenine-induced CKD, significantly and markedly diminished the antioxidants measured [28, 35], an action that was significantly mitigated by either gum acacia or SE given alone, and even more when combined. We have previously shown that SE did not affect the salutary action of another nephroprotective agent on renal histology, but it partially improved some of the measured biochemical and physiological analytes, suggesting that application of this type of exercise to treatment may improve further the benefits of the nephroprotective agents [18].

In the present work (Table 4), adenine was found to significantly increase the activities of plasma enzymes indicative of tissue damage (viz, ALT, ALP, GGT and AST). Interestingly, it has recently been reported that the liver histology was normal in an adenine-induced model of CKD, while the activities of two plasma enzymes, ALT and AST showed that the activities of these enzymes had been significantly inhibited four- and two-fold, respectively [51]. Contrary to these data we have found here, so have others, that the activities of these plasma enzymes are elevated and not inhibited by adenine [52–54]. SE did not significantly affect the activities of these enzymes, indicating that this moderate level of aerobic exercise has not caused significant tissue damage. Lisinopril and curcumin, given singly, did not cause significant effect on the activities of these enzymes. Combing each one of them in adenine-fed rats significantly ameliorated the rise in these plasma enzymes. This ameliorative effect was slightly but significantly enhanced further when both curcumin and lisinopril were given together to rats with CKD.

In conclusion, the present work has found salutary physiological, biochemical and histopathological actions of curcumin, lisinopril and SE, given separately to rats with adenine-induced CKD. However, administering these agents in combination may offer a histopathological benefit that is not afforded by SE. Further studies, using different experimental doses of the nephroprotective agents and exercise modalities are warranted to ascertain the influence of exercise on experimentally induced CKD.

## Supporting information

S1 File ARRIVE checklist. (PDF)
